# Koch’s Bacillus of Tubercle

**Published:** 1883-05-20

**Authors:** 


### *KOCH'S BACILLUS OF TUBERCLE.*

The discovery of Koch in relation to bacilli, as the cause of tubercular consumption, has been severely criticised on this side of the water, and many seemed ready to sneer at it as an unfortunate delusion; but Koch comes back vigorously at his criticisers, and is likely to sustain himself and make good his claim to one of the most wonderful and important discoveries of our day. The favorable results which may follow in the treatment of consumption, no one can foresee. The true cause of the malady being known, observations may now be hopefully directed to the discovery of proper and efficient therapeutic agencies.

